# Is it Time to Ditch the DICH (Delayed Intracranial Hemorrhage) Yet?

**DOI:** 10.7759/cureus.78047

**Published:** 2025-01-27

**Authors:** Emmanuel Luciano, Bianca Marquez, Akram Alashari, Narong Kulvatunyou

**Affiliations:** 1 Surgery, Central Michigan University College of Medicine, Saginaw, USA; 2 Thoracic Surgery, University of Miami, Miami, USA

**Keywords:** anticoagulation, anti platelet, ct (computed tomography) imaging, delayed intracranial hemorrhage, trauma

## Abstract

Background and objective

Delayed intracranial hemorrhage (DICH) is a well-known injury that is rare among patients who are on antiplatelet and/or anticoagulants with head trauma. In this study, we aimed to test the hypothesis that DICH is unlikely to occur without a history of head impact and signs of head and/or face trauma.

Materials and methods

We conducted a two-year (2020-2021) retrospective study regarding the incidence of DICH at our institution. During the study period, our institution had created a protocol specifically for this patient population who possibly suffered head trauma while taking anticoagulants and/or antiplatelets. The primary outcome was the incidence of DICH. The secondary outcome was the association between DICH and signs of head and/or facial trauma. The study protocol was reviewed and approved by the institutional review board committee.

Results

During the study period, there were 259 patients who had suffered head impact while taking anticoagulants and/or antiplatelets. Of them, 225 patients (86.9%) had a negative initial head CT and were admitted for observation. Repeat CT head was performed in 217 patients (96.4%). Among the patients who received a second CT head, only one patient (0.46%) had DICH. The patient did well clinically and did not require any neurosurgical intervention.

Conclusions

Our findings suggest that DICH in patients taking anticoagulants/antiplatelets is unlikely, and a repeat head CT is unnecessary in the absence of changes in neurological status. Thus, it can be concluded that eliminating the routine repeat second CT scan can decrease the length of hospital stay and hospitalization costs. This study's findings highlight the need for more appropriate training related to patients with a history of head impact and signs of head trauma.

## Introduction

A significant number of patients in the United States suffer from traumatic brain injury while taking anticoagulant/antiplatelet medications. The standard evaluation of these patients includes a CT scan of the head to assess for intracranial hemorrhage (ICH). However, if the initial CT scan is negative without any signs of head trauma, the suspicion of delayed intracranial hemorrhage (DICH) is low, even in patients on anticoagulation and/or antiplatelet medications. Moreover, the correlation between intracranial complications and different antithrombotic agents after head trauma is key in influencing the need for repeat imaging. Riccardi et al. in 2017 reported that patients taking novel oral anticoagulants (NOACs) had a decreased incidence of intracranial bleeds compared to patients taking vitamin K antagonists [1]. A study performed the following year provides further data to indicate that NOACs have lower fatal bleeding rates than vitamin K antagonists [2]. This is important as the consistency of the guidelines as to which antithrombotic agents warrant repeat imaging after a negative initial head CT is variable [3,4,5].

Some facilities have created protocols for the management of this patient population, which involves obtaining repeat CT scans of the head, performing serial neurological examinations, as well as keeping patients under 24-hour observation. While the incidence of DICH in patients on anticoagulation and/or antiplatelets is low, it still occurs and has a reported incidence range of 0-6% [6]. There is evidence to show that repeat imaging can diagnose DICH; however, the time interval for the development of DICH is highly variable, ranging from hours to up to five days [7]. The purpose of this study was to determine the incidence of DICH and evaluate the need for a repeat head CT to screen for DICH following head trauma in patients taking anticoagulants and/or antiplatelet medications.

## Materials and methods

Study design

We performed a retrospective analysis of trauma patients presenting following head trauma while taking anticoagulant and/or antiplatelet medications at a level 2 trauma center between 2020 and 2021. A cohort of 259 patients was identified. The inclusion criteria were as follows: individuals aged 18 years old and older on pre-admission prescribed anticoagulant and/or antiplatelet medications who had sustained head trauma. Patients who had an initial positive head CT scan were excluded. All patient interactions were reviewed from documentation in the electronic medical records. The protocol established by our trauma center during these two years was as follows: all patients on anticoagulation/antiplatelet with head trauma must be admitted to the trauma service along with performing a routine repeat CT head within a 12 to 24-hour time interval. The study protocol was reviewed and approved by the institutional review board (IRB) committee (protocol number: RMI20220123).

Statistical analysis

The baseline data analyzed comprised patient demographics and clinical presentation. Demographics included age and gender. Clinical presentation included type of anticoagulation/antiplatelet, International normalized ratio (INR), Glasgow Coma Scale (GCS) score, mechanism of injury, and injury severity score (ISS). Follow-up data included a repeat CT of the head and the duration between the initial and repeat CT head, ICU admission, and length of stay. Clinical outcomes were as follows: the incidence of DICH and the association between DICH and signs of head trauma. Data analysis was performed using Microsoft Excel version 16.86. Data are represented as means or frequencies with their corresponding percentages. No statistical tests to determine the significance were performed. All data are represented in a tabulated format. This work has been reported in line with the STROCSS criteria [8].

## Results

**Figure 1:**
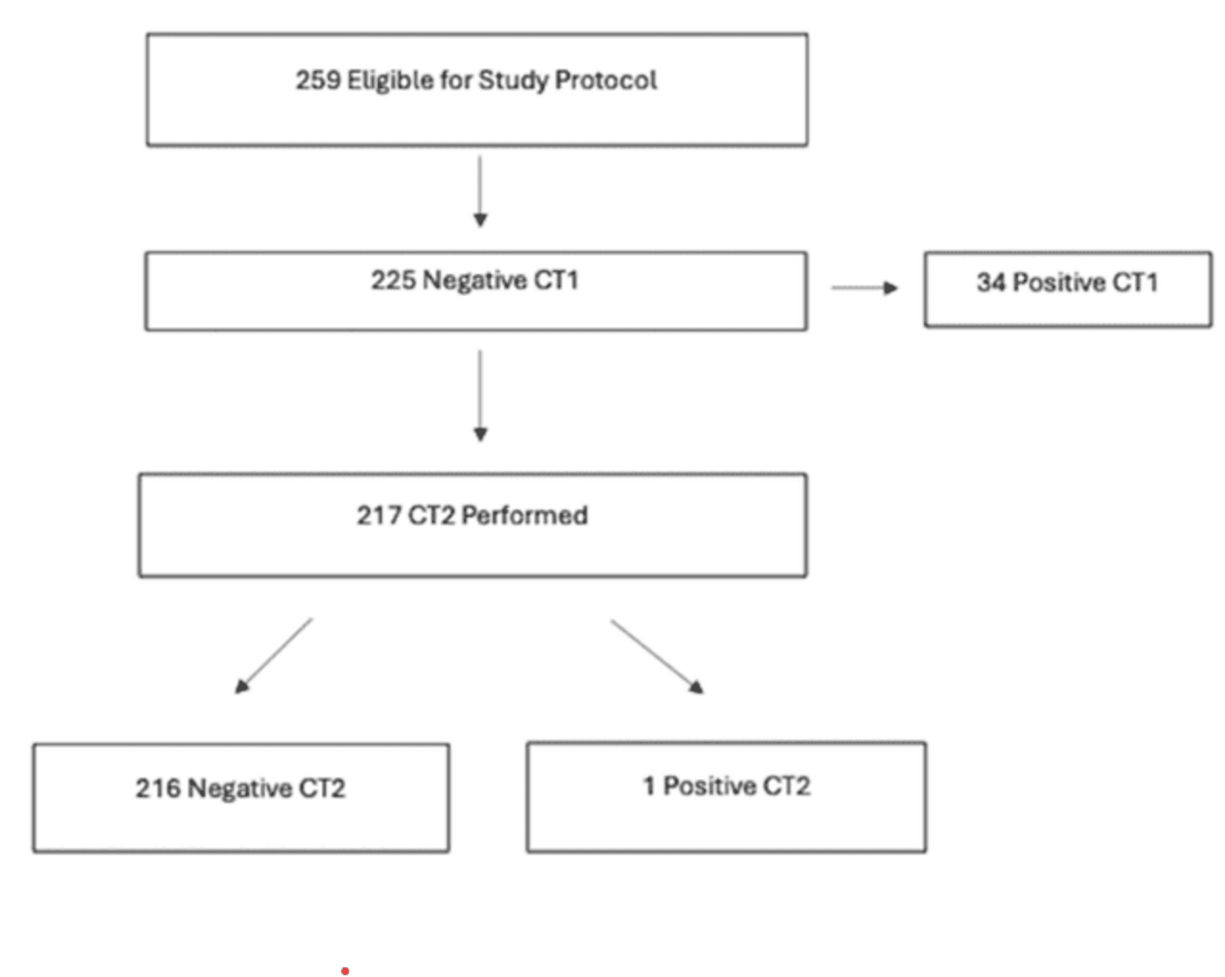
Patients included in the analysis CT1: first computed tomography; CT2: second computed tomography

**Figure 2:**
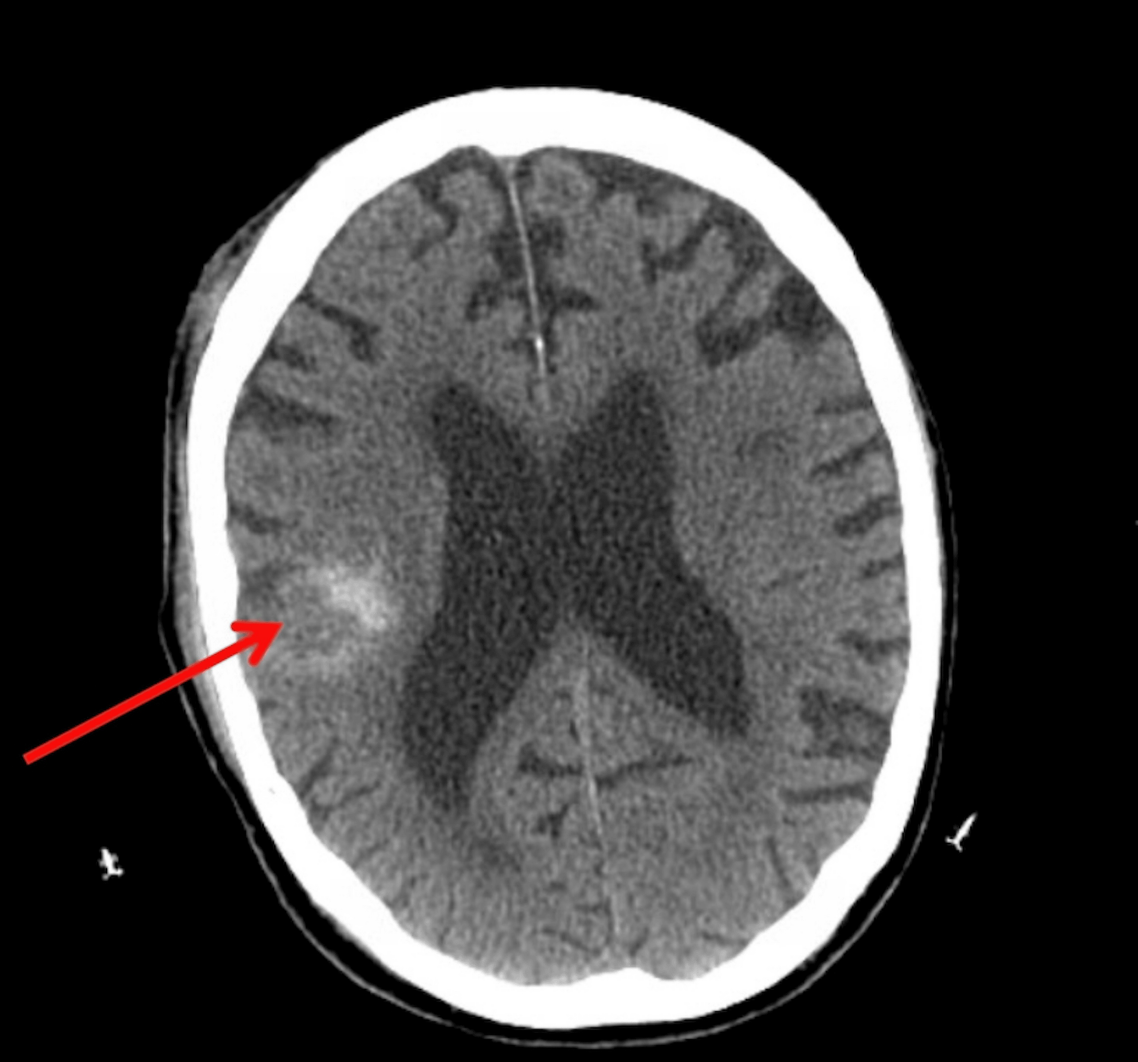
Repeat CT head of a 73-year-old male on clopidogrel demonstrating an intraparenchymal hemorrhage (arrow) CT: computed tomography

During the study period, 259 patients on anticoagulation and/or antiplatelet medication sustained head trauma. In 225 (86.9%) patients, the initial head CT scan was negative for intracranial injury, and, per protocol, they were admitted for observation and repeat head CT. The average time interval between the initial and the repeat head CT scan was 13.5 hours. Repeat CT head was performed in 217 patients (96.4%). Only one patient with a negative initial CT had DICH (0.46%) (Figure 1): a 73-year-old male on clopidogrel who sustained a fall with signs of head trauma and had a loss of consciousness. The repeat CT head revealed a newly developed intra-parenchymal and subarachnoid hemorrhage (Figure 2).

The patient did well clinically and did not require any neurosurgical intervention. The mechanism of injury for the majority of the cohort was mechanical fall (97.3%) as detailed in Table 1. The majority of the patients (138, 53.3%) were on NOACs. Approximately 26.6% of patients were on a combination of agents such as aspirin/clopidogrel, aspirin/warfarin, and aspirin/NOACs. Only two patients (0.77%) were on either Lovenox or dabigatran (Table 2). 

**Table 1:** Demographics and patient characteristics CT: computed tomography; DICH: delayed intracranial hemorrhage; GCS: Glasgow Coma Scale; ICU: intensive care unit; MVC: motor vehicle collision; SD: standard deviation

Characteristic	Values
Average age, years, mean (SD)	76.7 (29.6)
Males, n (%)	129 (49.8)
Females, n (%)	130 (50.2)
Fall, n (%)	252 (97.3)
MVC, n (%)	2 (0.77)
Self-inflicted, n (%)	2 (0.77)
Assault, n (%)	1 (0.39)
GCS score 3-8, n (%)	1 (0.39)
GCS score 13-14, n (%)	15 (5.8)
GCS score 15, n (%)	243 (93.8)
Negative CT 1, n (%)	225 (86.9)
Positive CT 1, n (%)	34 (13.1)
DICH, n (%)	1 (0.46)
CT 2 performed, n (%)	217 (96.4)
The interval between CT 1 and CT 2, hours, mean	13.5
Hospital length of stay, mean	5.0
ICU admission, n (%)	59 (22.8)

**Table 2:** The use of antiplatelets and/or anticoagulation agents in patients with negative initial CT CT: computed tomography; NOAC: novel oral anticoagulant

Antiplatelet/anticoagulation agents	N (%)
Aspirin only	0 (0%)
Clopidogrel only	13 (5%)
Warfarin only	37 (14.3%)
NOAC (apixaban, rivaroxaban)	138 (53.3%)
Other	2 (0.77%)
Combination	69 (26.6%)

## Discussion

Traumatic brain injury has been associated with worse outcomes in the setting of anticoagulation/antiplatelet use. Various studies have shown that obtaining a repeat CT head is not necessary in the evaluation for DICH, especially in the absence of signs of head and/or facial trauma. 

Kaen et al. prospectively analyzed patients on anticoagulation who suffered a mild traumatic brain injury - defined as GCS scores 13-15 - who had a negative initial head CT scan upon admission. All patients underwent a repeat head CT scan before discharge. Upon data analysis, only two patients out of their cohort had a new ICH; however, these patients required no further intervention [9]. In 2011, Peck et al. determined that the incidence of DICH was quite low and that the subjects with detected DICH did not require neurosurgical intervention. Similar to these findings, a study conducted in 2018 retrospectively analyzing elderly individuals who sustained a minor fall along with a subset of the cohort taking anticoagulants concluded that repeating head CT for detection of DICH is not worthwhile as overutilization of CT can lead to more financial burden, increased hospital stay, and higher radiation exposure [10,11]. In line with our data in 2020, out of 346 patients on oral anticoagulation, the incidence of DICH was quite low (2.3%), and those few that did develop DICH required no neurosurgical intervention. This again showed that performing another CT in the setting of a negative initial head CT is not warranted [12,13]. 

The findings of our retrospective study of 259 patients on preexisting anticoagulation/antiplatelet agents are consistent with the prior studies and highlight the importance of an efficient way to diagnose DICH. Guidelines such as the brain injury guidelines (BIG) have been proposed. The BIG guidelines are drawn from a large multicenter prospective study in 2022 [14]. Our findings amplify the current idea that all patients with head trauma with an initial negative head CT should not undergo repeat imaging unless certain factors, such as significant neurological decline, are present. When a repeat head CT scan is performed, it is most commonly done within 24 hours of the first CT scan. However, upon reviewing other studies such as those by Chenoweth et al. and Nishijima et al., the development of DICH can occur after 24 hours [7,15]. Instituting a protocol that standardizes a repeat CT within 24 hours of the first negative imaging may not help discover a majority of DICH incidents [16]. 

Our study has several limitations. Firstly, its retrospective design considerably hindered our ability to analyze causality. Second, our sample population comes from a single institution, and hence the external validity of our data is limited. However, despite these limitations, our study suggests that the development of DICH is rare in patients on anticoagulant/antiplatelet medications with an initial negative head CT scan.

## Conclusions

Reviews of the literature have challenged the utility of a repeat head CT scan in the setting of head trauma in patients taking anticoagulant and/or antiplatelet medications as the incidence of DICH is rare. In this study, we found that DICH does occur, and we should consider the history of head impact and signs of head trauma when triaging this patient population. Furthermore, we emphasize the need for a multi-center, prospective study to gain deeper insights into the topic and validate our findings on a broader scale.
